# Supplementary material for: DrugSniper, a Tool to Exploit Loss-Of-Function Screens, Identifies CREBBP as a Predictive Biomarker of VOLASERTIB in Small Cell Lung Carcinoma (SCLC)
Source: Cancers (Basel). 2020 Jul 7;12(7):1824. doi: 10.3390/cancers12071824 (PMC7408696; doi:10.3390/cancers12071824)
Supplement: Supplementary file 1 [file cancers-12-01824-s001.zip › cancers-835150 Supplementary layout/cancers-835150 Supplementary Section S1-S5.docx]

Supplementary Materials

DrugSniper, a Tool to Exploit Loss-of-Function Screens, Identifies *CREBBP* as a Predictive Biomarker of VOLASERTIB in Small Cell Lung Carcinoma (SCLC).

Fernando Carazo, Cristina Bértolo, Carlos Castilla, Xabier Cendoya, Lucía Campuzano, Diego Serrano, Marian Gimeno, Francisco J. Planes, Ruben Pio, Luis M. Montuenga and Angel Rubio

1. Supplementary Case Study—Lung Adenocarcinoma

As stated in the main manuscript, Drugsniper is capable of identifying not only loss-of-function but also gain-of-function mutations as predictive biomarkers of genetic essentiality. As a complementary analysis that supports this statement, we show here a case of study using lung adenocarcinoma, in which DrugSniper identifies a gain-of-function mutation to be a predictive biomarker. We applied DrugSniper to Lung-Adenocarcinoma cell lines (*n* = 42; Figure S1).


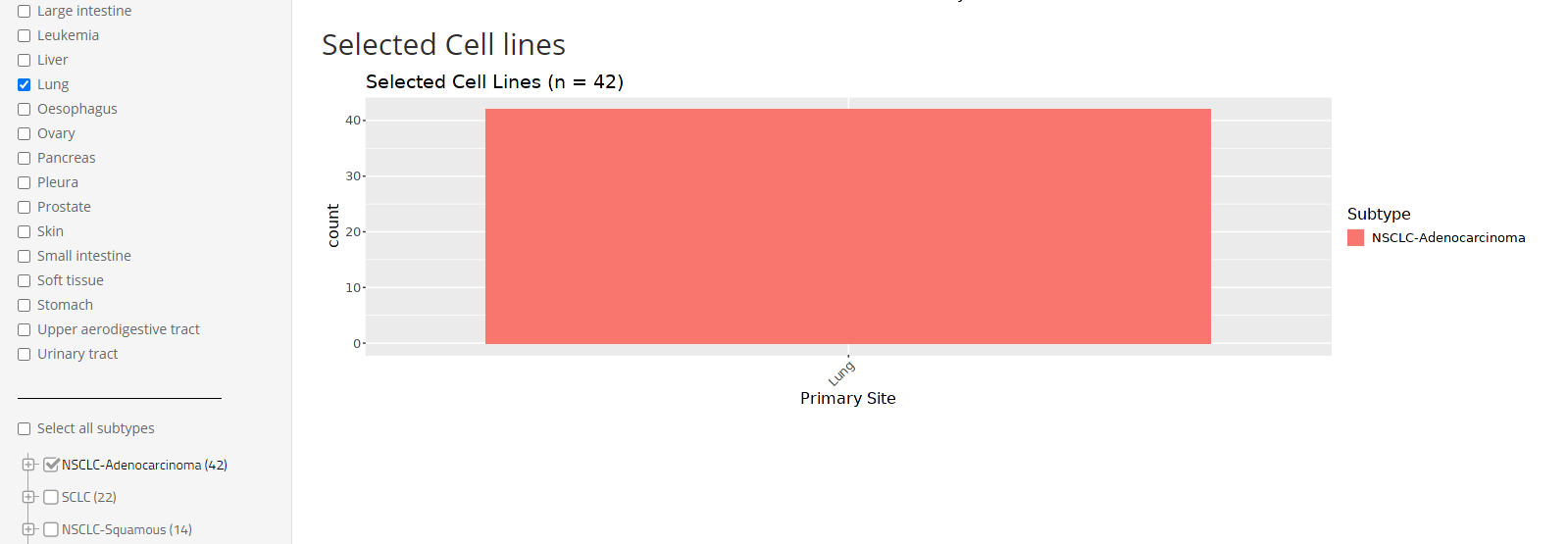


**Figure S1.** DrugSniper’s summary of lung adenocarcinoma selected cell lines (*n* = 42).

Moving on to Tab “2. Predict Biomarkers for a Target Gene” we run the analysis in which we did not have *a priori* selected drugs or targets to test: “All Protein Targets (558) & Drugs & Mutations” are set with the default filters. The goal was to obtain a list of putative drugs/targets with their corresponding predictive mutation biomarkers.

Using these three criteria, 558 targets were initially found to be essential and specific to lung adenocarcinoma cell lines. For the final list of hypotheses (Table 1), we required that the members of each pair of target and response biomarkers were also functionally/biologically related in the STRING protein-protein interactions database (an option also implemented in DrugSniper).

**
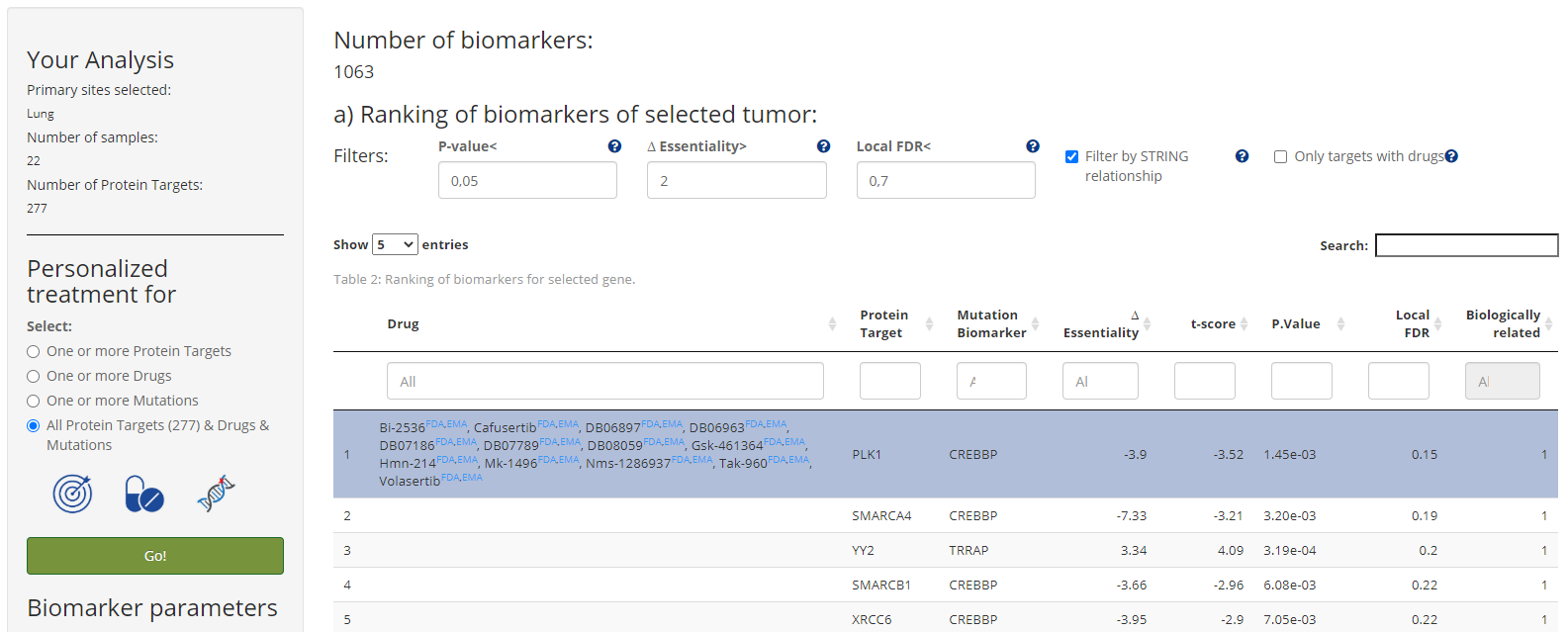
Figure S2.** DrugSniper’s snapshot of the ranking of predictive biomarkers using lung adenocarcinoma cell lines.

Using the above-mentioned filters, DrugSniper predicted 28 putative protein targets with their corresponding predictive mutation biomarkers that are significantly related to lung adenocarcinoma.

DrugSniper identifies 1,030 target-biomarker pairs that are significantly relevant in lung adenocarcinoma, 38 out of which have a biological interaction annotated in the String database (Table S3). Interestingly, the ranking includes *KRAS-KRASmut* pair (Top 1 pair when filtering by String), which is a well-known oncogene addition, triggered by a gain-of-functionality of *KRAS* due to its activating mutation.

**Table S4.** Ranking of drug targets and associated predictive mutation biomarkers (n = 38) for lung adenocarcinoma cell lines using DrugSniper.

| Drug | Protein Target | Mutation Biomarker | Variation in Essentiality | t-*Score* | *P-*Value | Local FDR |
| --- | --- | --- | --- | --- | --- | --- |
| DB07771, FARNESYL DIPHOSPHATE | KRAS | KRAS | −4.42 | −5.71 | 5.88 × 10^−7^ | 0 |
| NA | SRSF1 | DYRK1A | −2.22 | −5 | 1.56 × 10^−2^ | 0.11 |
| NA | LONP1 | HSP90B1 | −3.6 | −3.34 | 1.56 × 10^−3^ | 0.13 |
| NA | MYL12B | MYLK | 4.65 | 2.94 | 4.88 × 10^−3^ | 0.13 |
| NA | RANBP2 | HSP90B1 | −4.45 | −3.23 | 2.19 × 10^−3^ | 0.14 |
| NA | RPS6 | HSP90B1 | −2.5 | −3.21 | 2.31 × 10^−3^ | 0.14 |
| NA | TOMM40 | HSP90B1 | −4.34 | −2.96 | 4.64 × 10^−3^ | 0.17 |
| NA | PSMC4 | HSP90B1 | −2.44 | −2.55 | 1.37 × 10^−2^ | 0.19 |
| NA | EIF3A | HSP90B1 | −2.62 | −2.35 | 2.25 × 10^−2^ | 0.21 |
| NA | DYNC1H1 | DNAH8 | −2.55 | −2.23 | 3.04 × 10^−2^ | 0.22 |
| NA | VARS | HSP90B1 | −2.14 | −2.15 | 3.65 × 10^−2^ | 0.23 |
| NA | FAU | HSP90B1 | −2.93 | −2.12 | 3.89 × 10^−2^ | 0.23 |
| NA | SMARCA4 | HSP90B1 | −3.83 | −2.12 | 3.91 × 10^−2^ | 0.23 |
| NA | HSPD1 | HSP90B1 | −2.77 | −2.04 | 4.71 × 10^−2^ | 0.24 |
| NA | ATP2A2 | EGFR | 2.56 | 2.91 | 5.31 × 10^−3^ | 0.26 |
| N-Formylmethionine | UBC | EPHA5 | −3.15 | −2.55 | 1.38 × 10^−2^ | 0.29 |
| NA | HNRNPK | EGFR | 2.05 | 2.61 | 1.19 × 10^−2^ | 0.3 |
| Bortezomib, Carfilzomib | PSMA7 | EGFR | 2.84 | 2.59 | 1.26 × 10^−2^ | 0.31 |
| NA | RPF2 | EIF4E | −3.06 | −3.78 | 4.18 × 10^−4^ | 0.32 |
| NA | MED12 | EP300 | −4.91 | −3.49 | 1.02 × 10^−3^ | 0.33 |
| NA | HEATR1 | TTBK1 | 2.05 | 2.04 | 4.62 × 10^−2^ | 0.4 |
| NA | MCM7 | EGFR | 3.09 | 2.09 | 4.13 × 10^−2^ | 0.41 |
| NA | RPN1 | EGFR | 2.25 | 2.05 | 4.51 × 10^−2^ | 0.42 |
| N-Formylmethionine | UBC | TRIM33 | 2.62 | 2.55 | 1.39 × 10^−2^ | 0.42 |
| NA | SMC2 | EGFR | 2.28 | 2.03 | 4.81 × 10^−2^ | 0.43 |
| NA | CSE1L | EGFR | 2.76 | 2.01 | 4.99 × 10^−2^ | 0.43 |
| NA | USP7 | TP53 | 3.34 | 3.31 | 1.71 × 10^−3^ | 0.46 |
| NA | SLBP | EIF4E | −2.97 | −3.08 | 3.34 × 10^−3^ | 0.48 |
| NA | EIF3F | EIF4E | −2.19 | −2.78 | 7.61 × 10^−3^ | 0.49 |
| NA | COPS3 | TP53 | 2.74 | 2.3 | 2.57 × 10^−2^ | 0.52 |
| NA | RPS12 | EIF4E | −2.52 | −2.47 | 1.68 × 10^−2^ | 0.53 |
| NA | ZNF189 | PDGFRA | −2.56 | −2.55 | 1.37 × 10^−2^ | 0.59 |
| NA | EIF3A | EIF4E | −2.26 | −2.16 | 3.52 × 10^−2^ | 0.59 |
| NA | EIF4G1 | EIF4E | −4.42 | −2.16 | 3.54 × 10^−2^ | 0.59 |
| NA | RAPGEF1 | PDGFRA | 2.96 | 2.3 | 2.57 × 10^−2^ | 0.59 |
| Azd-6738, BERZOSERTIB, Vx-970 | ATR | ATR | −2.02 | −2.58 | 1.30 × 10^−2^ | 0.59 |
| Alvocidib, Bms-387032 | CDK9 | ATR | −2.32 | −2.33 | 2.37 × 10^−2^ | 0.59 |
| AP1903, Apitolisib | MTOR | ATR | −2.66 | −2.16 | 3.58 × 10^−2^ | 0.62 |

The ranking is sorted according to the local FDR (adjusted p-value), more information in the Methods Section. The column ΔEss represents the average change in the DEMETER score between mutated and wild-type cells. If ΔEss < 0, mutated cell lines are sensitive to the inhibition of the drug target and wild-type cells are resistant; if ΔEss > 0, wild-type cells are sensitive and mutated cells resistant. The rest of the columns are (% Mut Patient) percentage levels of each mutation in patients; (*p*-value) statistical significance before adjusting; and (lfdr) local false discovery rate adjusted *p*-value. * Targets with an existing approved or experimental inhibitor molecule.


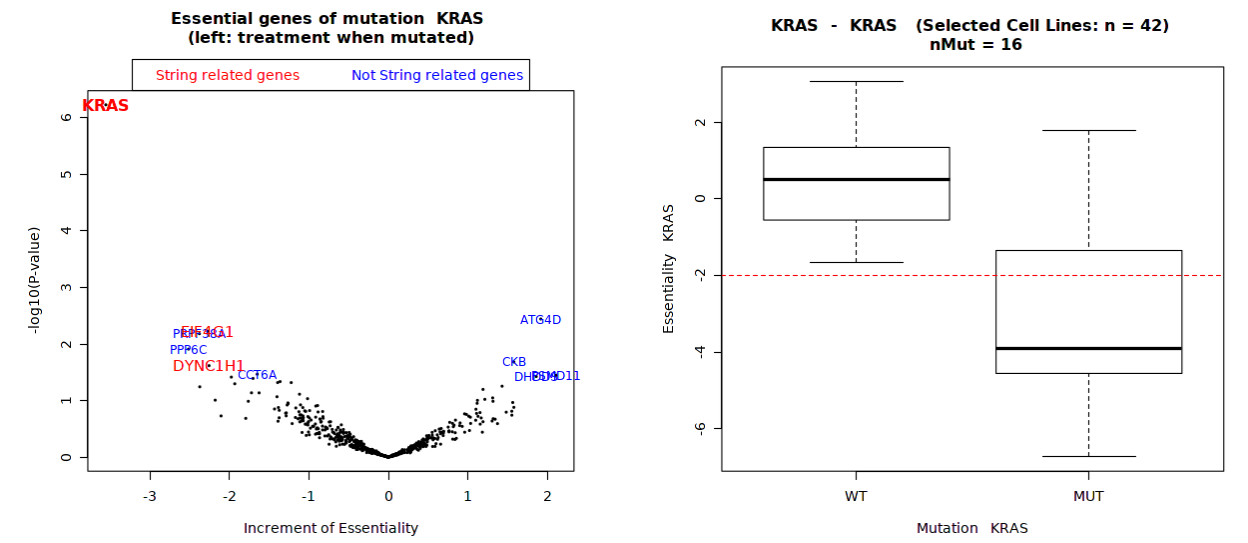


**Figure S3.** (Left) Volcano plot showing top essential genes that depend on the mutational status of KRAS. If KRAS is mutated, the KRAS gene is essential for lung adenocarcinoma cells’ viability, suggesting that KRAS is a good predictive biomarker of KRAS essentiality. (Right) Box plot showing KRAS’s essentiality score (DEMETER score) regarding KRAS mutational status. KRAS is essential for KRAS -mutant cell lines. The dotted red line represents the -2 DEMETER score threshold (which is the essentiality threshold proposed by DEMETER’s authors).

2. Literature Pairs Validation Pipeline

Olaparib (PARP1)-BRCA2 association was validated in “Visualize case-by-case” selecting all Primary sites and their corresponding histological subtypes (*p*-value 6.62 × 10^−3^local FDR 0.01). The essentiality score is lower (more sensitive) with mutant cells. However, the absolute level is above −2 that is the default threshold in “Predict Biomarkers for a Target Gene”


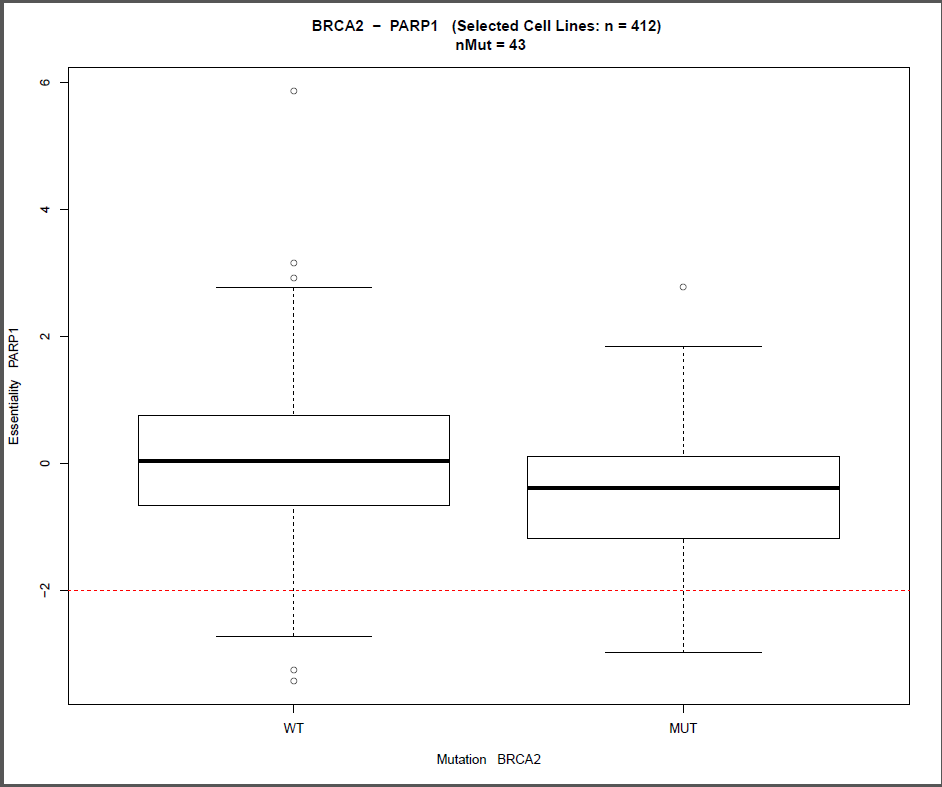


**Figure S4.** Box plot showing PARP1’s essentiality score (DEMETER score) regarding BRCA2 mutational status.

MAP3K7-ATM mutation was validated in the “Visualize case-by-case” tab and selecting NSCLC histological subtypes (*p*-value 5.44 × 10^−4^).


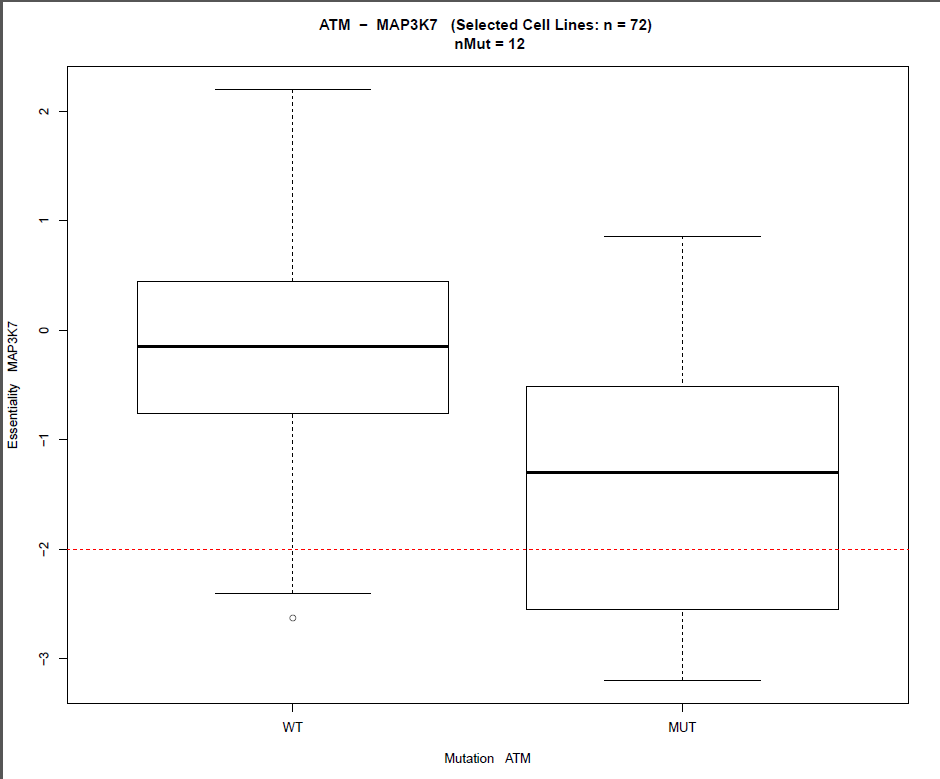


**Figure S5.** Box plot showing MAP3K7’s essentiality score (DEMETER score) regarding ATM mutational status.

EGFR-EGFR association was found by adding additional primary sites to the analysis to increase the statistical power: Lung, Pleura, Oesophagus, and Prostate cancers.


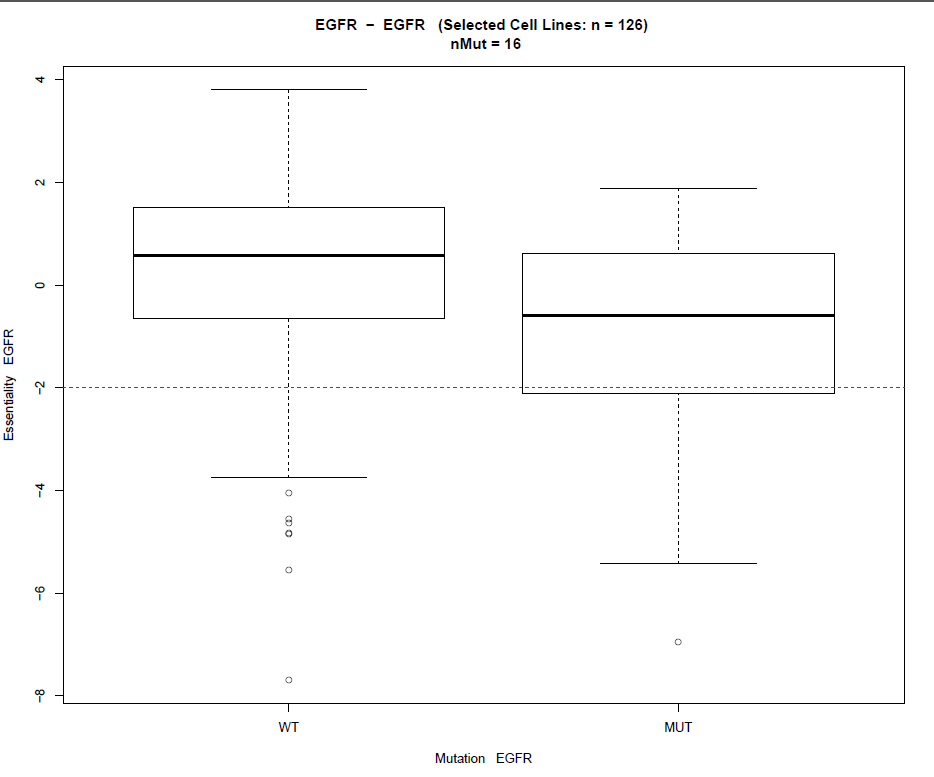


**Figure S6.** Box plot showing EGFR’s essentiality score (DEMETER score) regarding EGFR mutational status.

3. CCLE SCLC Gene Variant Description

We intended to identify the main *CREBBP* variants in SCLC cell lines. In this section, we give more details about SCLC gene variants of CCLE cancer cell lines (Figure S7), specifically *CREBBP* (Table S4). For this analysis, we have used the MAF version of CCLE cell lines CCLE_hybrid_capture1650_hg19_NoCommonSNPs_NoNeutralVariants_CDS_2012.05.07.maf, which includes a loss-of-function (LOF) mutations in ~1650 genes across CCLE cell lines.

Data used for the study was already pre-processed by CCLE authors. The following variants were filtered out by CCLE authors: common polymorphisms, allelic fraction < 10%, putative neutral variants (missenses present in less than 2 warm-blooded vertebrates), located outside of the CDS for all transcripts.

Remarkably, variants identified in *CREBBP* include missense, nonsense, and in-frame deletions (Figure S8; Figure S9), emphasizing the deleterious effect of these types of variants. Moreover, *CREBBP* variants appear not to be statistically significantly (*p*.value < 0.05) co-occurrent with other gene variants (Figure S10). This fact potentiates *CREBBP* has an idyllic biomarker, that clearly differentiates the cohort of samples sensitive to *PLK1*-inhibitors.


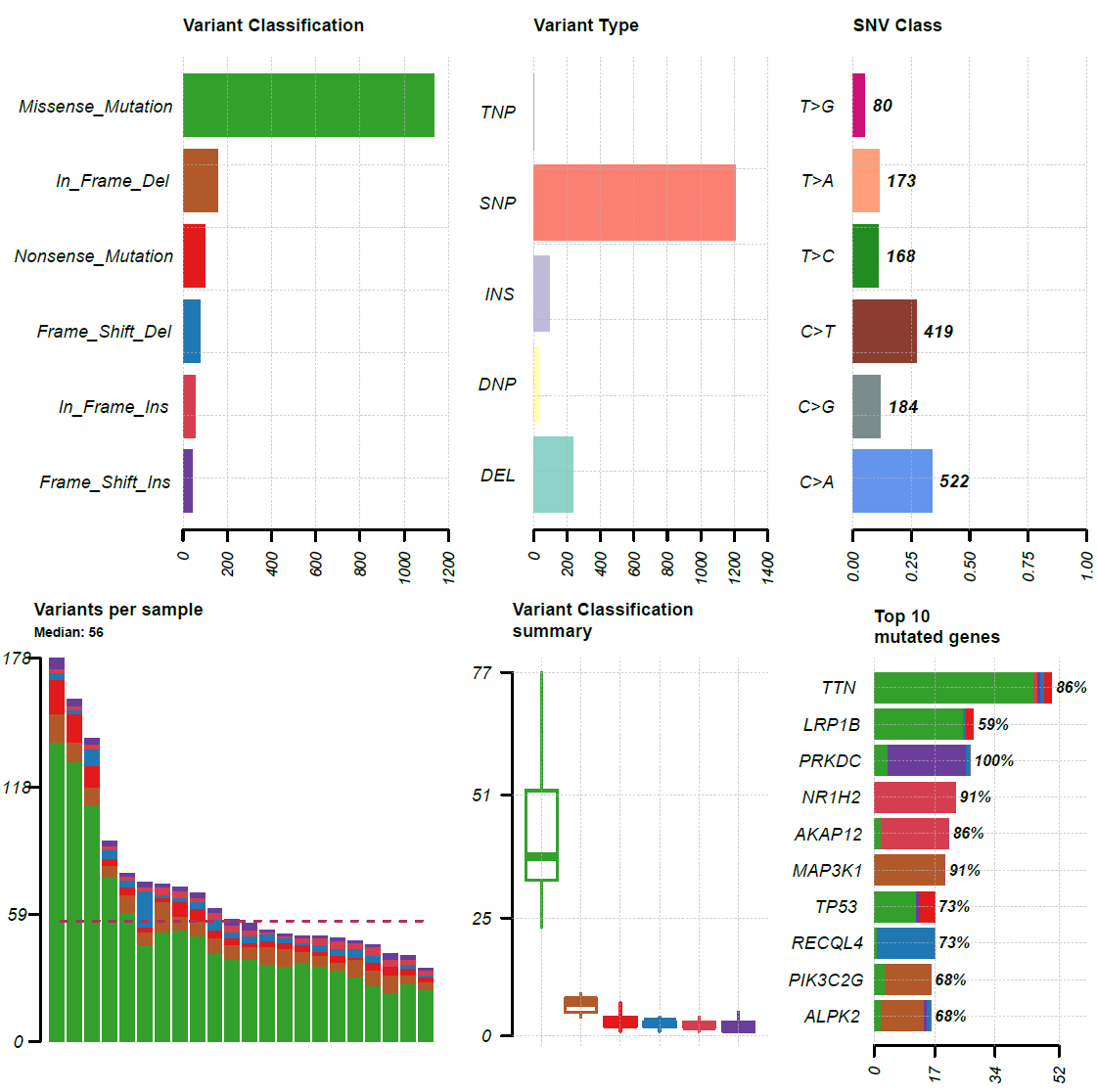


**Figure S7.** Gene variant information of SCLC CCLE cell lines (*n* = 22).


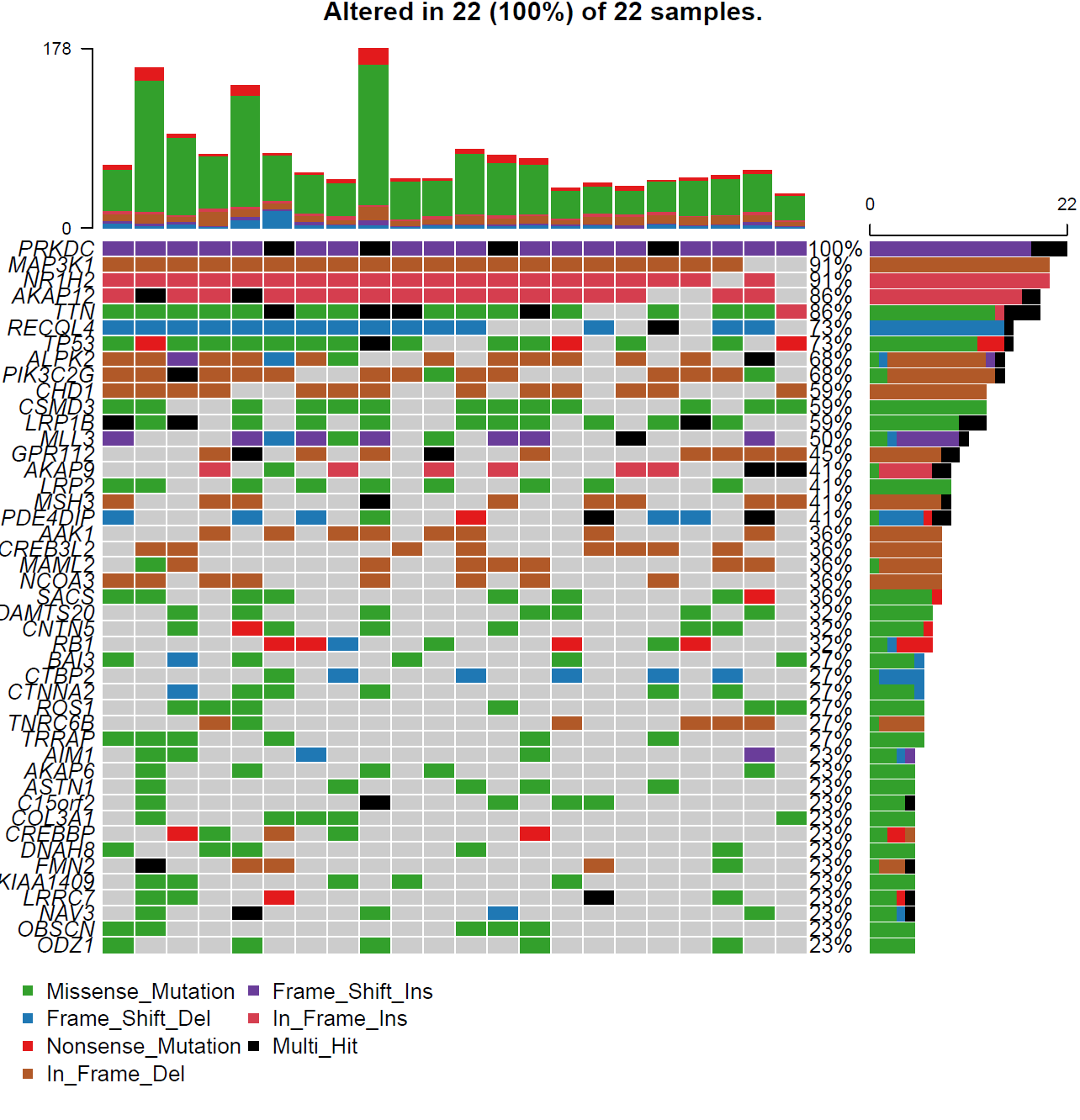


**Figure S8.** Oncoplot of SCLC CCLE cell lines.

**Table S5.** Information of Gene variants of SCLC CCLE cell lines.

| **Hugo_Symbol** | **Entrez_Gene_Id** | **End_Position** | **Strand** | **Variant_Classification** | **Variant_Type** | **Reference_Allele** | **Tumor_Seq_Allele1** |
| --- | --- | --- | --- | --- | --- | --- | --- |
| CREBBP | 3779578 | 3779578 | + | Missense_Mutation | SNP | C | T |
| CREBBP | 3781324 | 3781326 | + | In_Frame_Del | DEL | AGG | - |
| CREBBP | 3786075 | 3786075 | + | Missense_Mutation | SNP | T | C |
| CREBBP | 3808968 | 3808968 | + | Nonsense_Mutation | SNP | T | A |
| CREBBP | 3841985 | 3841985 | + | Nonsense_Mutation | SNP | G | A |
|  |  |  |  |  |  |  |  |
| **dbSNP_RS** | **Matched_Norm_Sample_Barcode** | **Genome_Change** | **Annotation_Transcript** | **Transcript_Strand** | **cDNA_Change** | **Codon_Change** | **Protein_Change** |
| HCC33_LUNG | Illumina GAIIx | g.chr16:3779578C>T | uc002cvv.2 | - | c.5470G>A | c.(5470-5472)GCC>ACC | p.A1824T |
| NCIH1048_LUNG | Illumina GAIIx | g.chr16:3781324_3781326delAGG | uc002cvv.2 | - | c.5039_5041delCCT | c.(5038-5043)TCCTTG>TTG | p.S1680del |
| NCIH211_LUNG | Illumina GAIIx | g.chr16:3786075T>C | uc002cvv.2 | - | c.4690A>G | c.(4690-4692)AAA>GAA | p.K1564E |
| NCIH1876_LUNG | Illumina GAIIx | g.chr16:3808968T>A | uc002cvv.2 | - | c.3256A>T | c.(3256-3258)AAA>TAA | p.K1086* |
| NCIH1105_LUNG | Illumina GAIIx | g.chr16:3841985G>A | uc002cvv.2 | - | c.1327C>T | c.(1327-1329)CAA>TAA | p.Q443* |
|  |  |  |  |  |  |  |  |
| **Refseq_mRNA_Id** | **Refseq_prot_Id** | **SwissProt_acc_Id** | **SwissProt_entry_Id** | **Description** | **UniProt_AApos** | **UniProt_Region** | **UniProt_Site** |
| NM_004380 | NP_004371 | Q92793 | CBP_HUMAN | CREB binding protein isoform a | 1824 | TAZ-type2.\|Interaction with TRERF1. | 16 |
| NM_004380 | NP_004371 | Q92793 | CBP_HUMAN | CREB binding protein isoform a | 1680 | Interaction with TRERF1. | 10 |
| NM_004380 | NP_004371 | Q92793 | CBP_HUMAN | CREB binding protein isoform a | 1564 | Interaction with TRERF1. | 79 |
| NM_004380 | NP_004371 | Q92793 | CBP_HUMAN | CREB binding protein isoform a | 1086 |  | 54 |
| NM_004380 | NP_004371 | Q92793 | CBP_HUMAN | CREB binding protein isoform a | 443 |  | 94 |


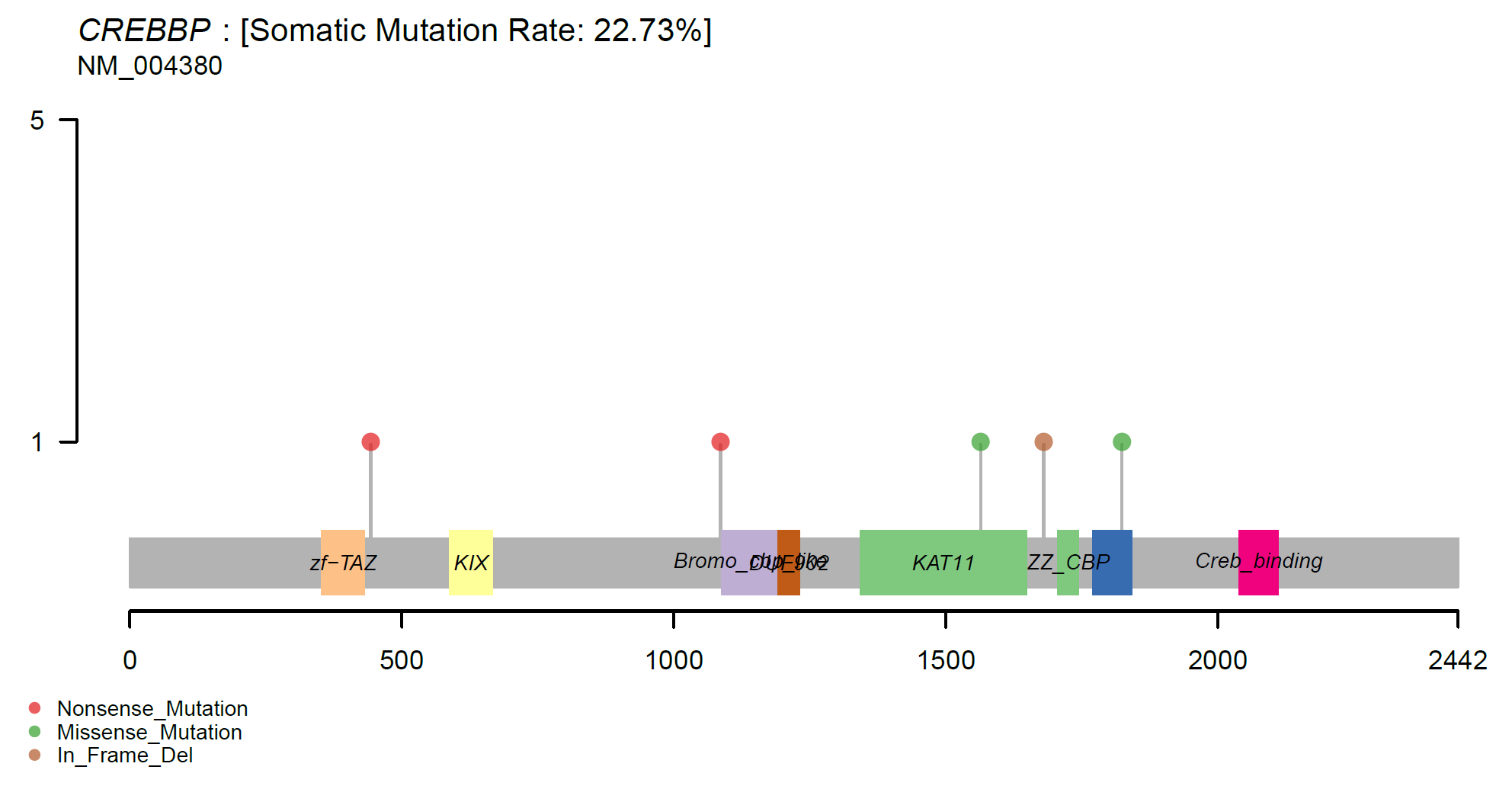


**Figure S9.** CREBBP Lollipop plot of amino acid changes on to protein structure in SCLC CCLE cell lines.


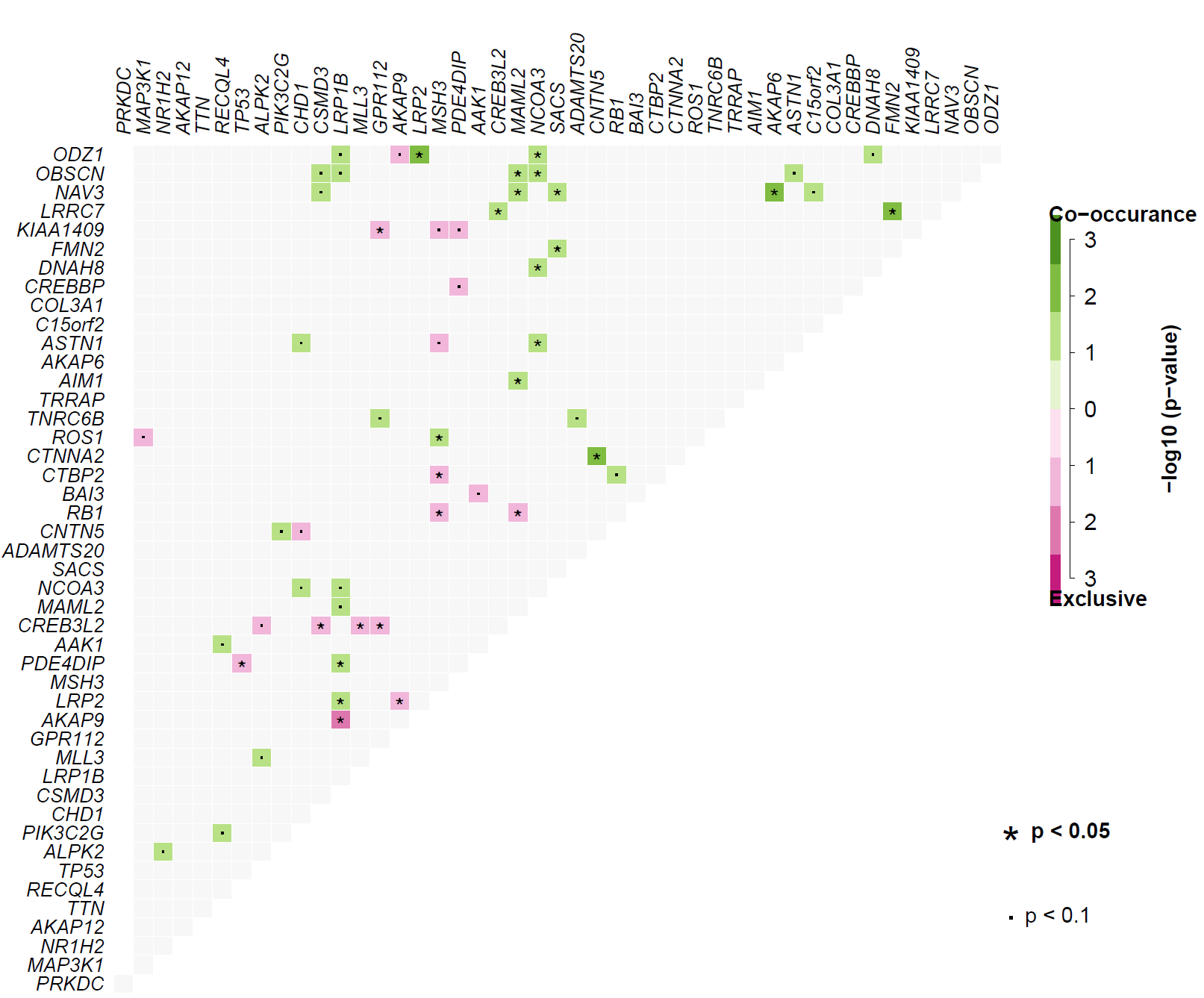


**Figure S10.** Gene variant interactions. Mutually exclusive, co-occurring, and altered genesets. Pair-wise Fisher's Exact test was done to detect mutually exclusive or co-occurring events. Also identifies gene sets mutated significantly. *p* < 0.1, **p* < 0.05.

4. Additional Validating Data of *PLK1* Inhibitors in SCLC Cell Lines

**Table S6.** Volasertib estimated effective doses.

| CELL LINE | IC50 | CREBBP status |
| --- | --- | --- |
| **e:H1048:50** | 5.4413 | MUT |
| **e:H146:50** | 11.6791 | WT |
| **e:H1963:50** | 1.8929 | MUT |
| **e:H211:50** | 4.7279 | MUT |
| **e:H2171:50** | 22.4232 | WT |
| **e:H841:50** | 15.6826 | WT |
| **e:H889:50** | 3.8152 | WT |
| **e:HCC33:50** | 5.2447 | MUT |

**Table S7.** BI2536 estimated effective doses.

| CELL LINE | IC50 | CREBBP status |
| --- | --- | --- |
| **e:H1048:50** | 2.414 | MUT |
| **e:H146:50** | 14.2914 | WT |
| **e:H1963:50** | NA | MUT |
| **e:H211:50** | 4.6315 | MUT |
| **e:H2171:50** | 9.7577 | WT |
| **e:H841:50** | 11.157 | WT |
| **e:H889:50** | 2.8038 | WT |
| **e:HCC33:50** | 6.0446 | MUT |


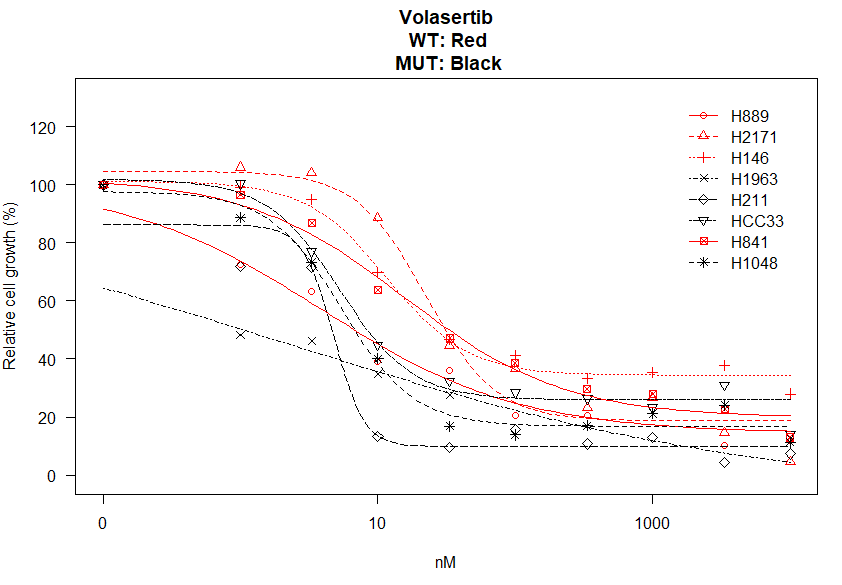


**Figure S11.** Dose-response curves showing the effect of Volasertib treatment on the viability of CREBBP-WT (red) NCI-H841, NCI-H889, NCI-H2171, NCI-H146 cells, and CREBBP-MUT (black) NCI-H1048, NCI-H1963, NCI-H211, HCC33 cells.


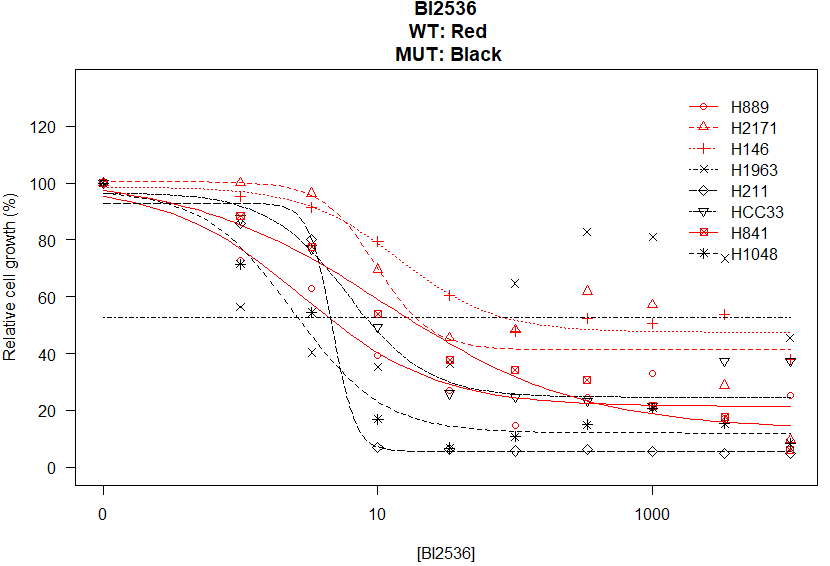


**Figure S12.** Dose-response curves showing the effect of BI2536 treatment on the viability of CREBBP-WT (red) NCI-H841, NCI-H889, NCI-H2171, NCI-H146 cells, and CREBBP-MUT (black) NCI-H1048, NCI-H1963, NCI-H211, HCC33 cells.

5. A Quick Start Guide for Using DrugSniper Application

5.1. Quick Start

DrugSniper outputs a ranking of putative drug target genes and their companion biomarkers for a given cohort of cell lines.

The functionalities of DrugSniper are presented in a set of panels:

5.1.1. Select Cell Lines

The user is required to select the cohort of cell lines to be analyzed. Several primary sites, subtypes, and cell lines can be selected at the same time. The application is pre-loaded with all the necessary data so that the user does not need to upload any data.


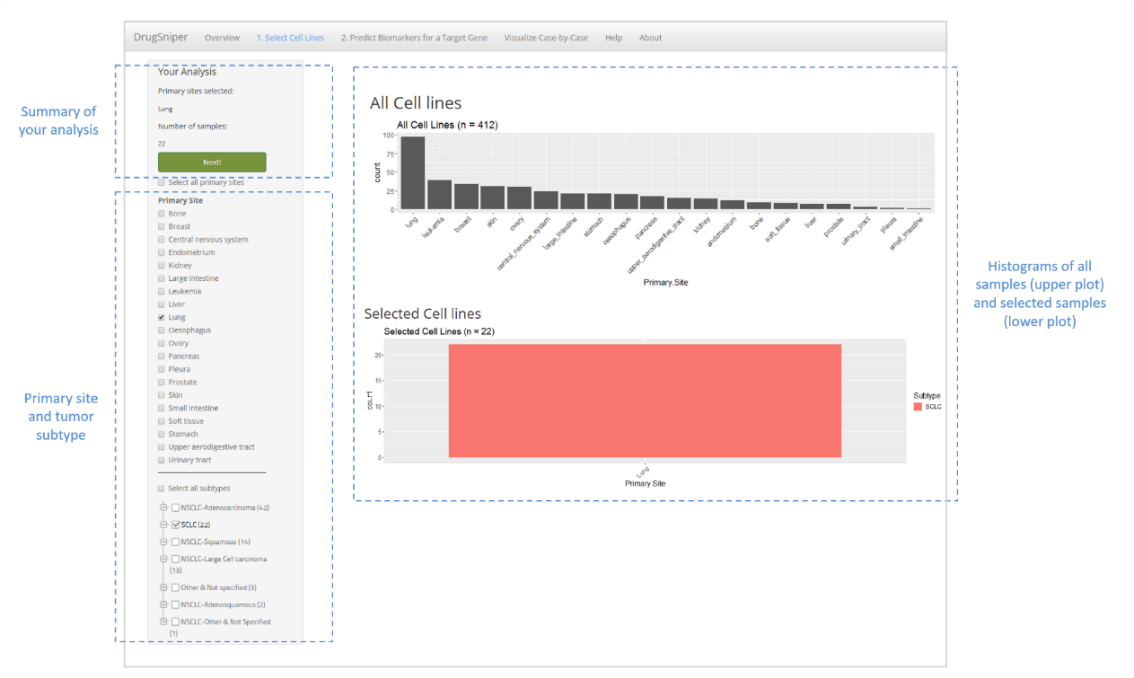


**Figure S13.** DrugSniper’s tab with a summary of selected cell lines.

5.1.2. Predict Biomarkers for a Target Gene

The second step of the analysis starts with the automatic extraction of the genes that are essential for cell viability of the selected cell lines. Although, if it is desired, five tunable parameters are given to the user to tailor the required threshold for a gene to be essential. By default, the toolbox already provides a value for each parameter which corresponds to our understanding of the minimum conditions which need to be satisfied by a gene to be essential.

The essentiality filters correspond to three criteria: essentiality, specificity, and expression. Essentiality is a two-legged characteristic. It refers to the percentage of selected cell lines that have a DEMETER score lower than the essentiality cut-off (see NOTE). Specificity is represented by the enrichment ratio. This filter allows the user to define the minimum ratio between the proportion of selected cell lines for which a gene is essential and the proportion of the rest of the cell lines for which the same gene is essential. Finally, essential genes are required to be expressed according to the expression filters.

Once the essential genes are automatically computed, the user can select one or more of these essential genes/protein targets and predict putative mutation biomarkers of essentiality. The user can also filter the results by one or more biomarkers or by one or more inhibition drugs related to the protein targets.


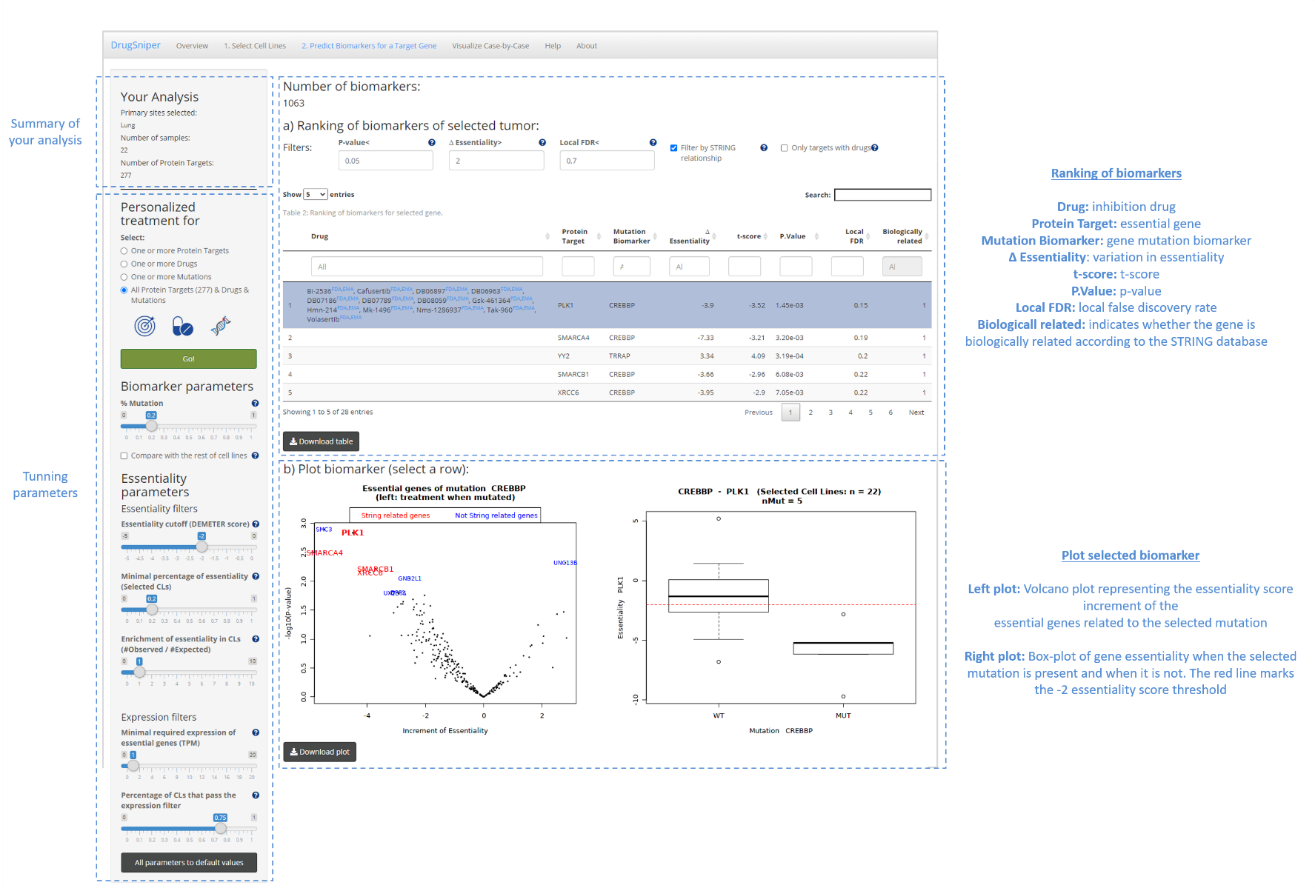


**Figure S14.** DrugSniper’s tab for predicting biomarkers for a target gene.

5.1.3. Visualize Case-by-Case

In this section, the user can select one of the essential genes/protein targets and a putative mutation biomarker of essentiality to get the pair statistical values.


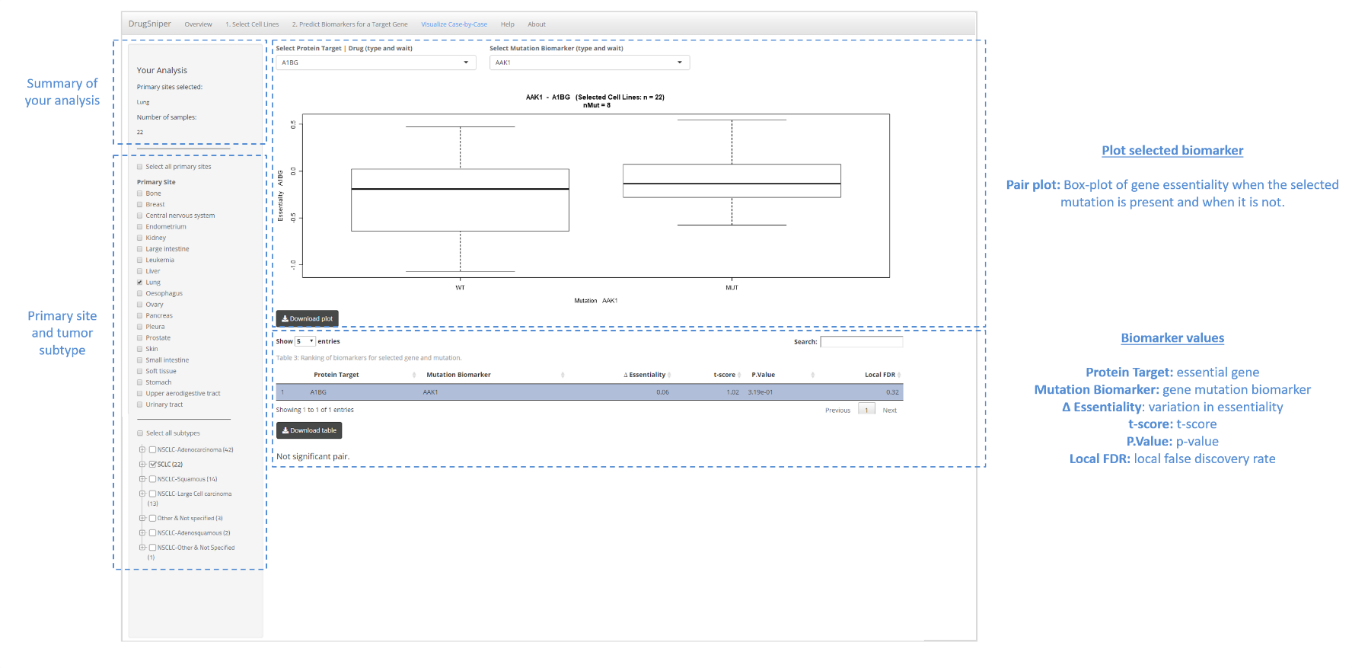


**Figure S15.** DrugSniper’s tab for visualizing case-by-case pairs of target gene-biomarker.

5.2. Running DrugSniper Locally

DrugSniper can also be run locally. The databases and source code are available at [GitLab](https://gitlab.com/ccastilla.1/DrugSniper).

Once the git repository is cloned, the shiny app can be run locally simply by typing: shiny::runApp('./app.R')

5.3. Data Integration and Workflow

The Cancer Cell Line Encyclopedia (CCLE) provides public access to genomic data of up to 900 cancer cell lines.

Project Achilles includes RNAi loss-of-function screens of over 17,000 genes in around 500 cancer cell lines. 568 of these cell lines match CCLE’s cell line cohort.

More details of materials and methods can be found in the main manuscript.


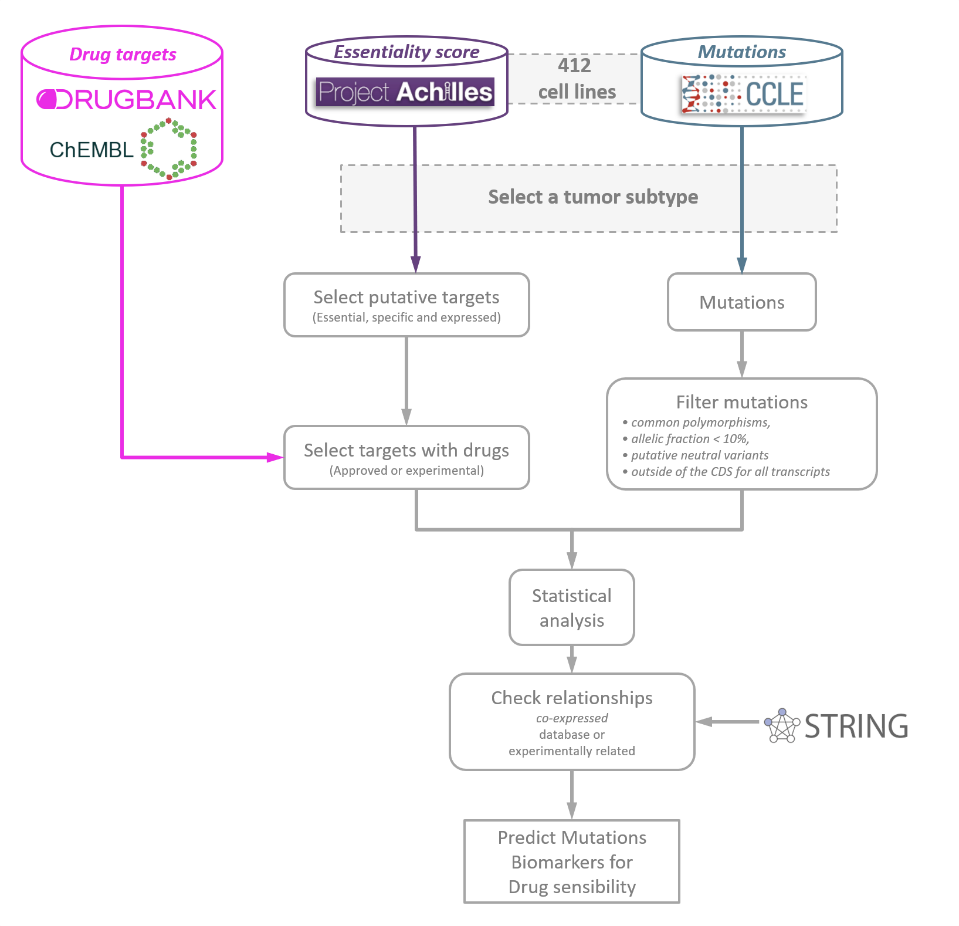


**Figure S16.** Scheme of the DrugSniper’s processing pipeline.

5.4. Note.

DrugSniper uses the DEMETER score as the essentiality score. The more negative the DEMETER score is, the more essential the gene is for a cell line.

Authors of DEMETER established a cut-off of -2 as a threshold of essentiality. Genes with DEMETER scores lower than this threshold can be considered essential for a cell line.

| 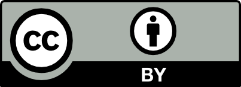 | © 2020 by the authors. Licensee MDPI, Basel, Switzerland. This article is an open access article distributed under the terms and conditions of the Creative Commons Attribution (CC BY) license (http://creativecommons.org/licenses/by/4.0/). |
| --- | --- |
